# Portable sequencing of *Mycobacterium tuberculosis* for clinical and epidemiological applications

**DOI:** 10.1093/bib/bbac256

**Published:** 2022-07-27

**Authors:** Paula J Gómez-González, Susana Campino, Jody E Phelan, Taane G Clark

**Affiliations:** Faculty of Infectious and Tropical Diseases, London School of Hygiene & Tropical Medicine, WC1E 7HT London, UK; Faculty of Infectious and Tropical Diseases, London School of Hygiene & Tropical Medicine, WC1E 7HT London, UK; Faculty of Infectious and Tropical Diseases, London School of Hygiene & Tropical Medicine, WC1E 7HT London, UK; Faculty of Infectious and Tropical Diseases, London School of Hygiene & Tropical Medicine, WC1E 7HT London, UK; Faculty of Epidemiology and Population Health, London School of Hygiene & Tropical Medicine, WC1E 7HT London, UK

**Keywords:** Mycobacterum tuberculosis, tuberculosis, sequencing, genomics, mutations

## Abstract

With >1 million associated deaths in 2020, human tuberculosis (TB) caused by the bacteria *Mycobacterium tuberculosis* remains one of the deadliest infectious diseases. A plethora of genomic tools and bioinformatics pipelines have become available in recent years to assist the whole genome sequencing of *M. tuberculosis*. The Oxford Nanopore Technologies (ONT) portable sequencer is a promising platform for cost-effective application in clinics, including personalizing treatment through detection of drug resistance-associated mutations, or in the field, to assist epidemiological and transmission investigations. In this study, we performed a comparison of 10 clinical isolates with DNA sequenced on both long-read ONT and (gold standard) short-read Illumina HiSeq platforms. Our analysis demonstrates the robustness of the ONT variant calling for single nucleotide polymorphisms, despite the high error rate. Moreover, because of improved coverage in repetitive regions where short sequencing reads fail to align accurately, ONT data analysis can incorporate additional regions of the genome usually excluded (e.g. *pe*/*ppe* genes). The resulting extra resolution can improve the characterization of transmission clusters and dynamics based on inferring closely related isolates. High concordance in variants in loci associated with drug resistance supports its use for the rapid detection of resistant mutations. Overall, ONT sequencing is a promising tool for TB genomic investigations, particularly to inform clinical and surveillance decision-making to reduce the disease burden.

## Introduction

*Mycobacterium tuberculosis* remains one of the deadliest single infectious agents, leading to 10 million human tuberculosis (TB) cases and 1.5 million associated deaths in 2020 [1]. The *M. tuberculosis* complex is phylogeographically distributed in defined lineages that can determine the emergence of drug resistance, transmissibility, pathogenicity and host response, disease site and severity [2–4]. Drug-resistant *M. tuberculosis* is one of the major threats to effectively controlling the disease, especially resistance to first-line rifampicin and isoniazid; in combination, called multi-drug resistance (MDR-TB). In fact, MDR-TB accounted for around 150 000 cases in 2020 [1]. The acquisition of drug resistance in *M. tuberculosis* has been mainly attributed to spontaneous mutations, such as single nucleotide polymorphisms (SNPs) and small insertions and deletions (indels) in genes coding for drug targets, drug-converting enzymes or loci involved in the transport of small molecules such as efflux pumps [5, 6]. Phenotypic susceptibility testing is the traditional method to determine drug resistance. In combination with genome-wide association and convergent evolution studies, genetic variants conferring drug resistance have been validated enabling the use of genotypic methods to establish resistance through sequencing or nucleic acid amplification approaches [5]. Transmission events can be inferred through the identification of *M. tuberculosis* isolates with (near) identical genomes (genomic variants), sourced from different individuals or patients [7]. Characterizing the phylogeographic distribution of *M. tuberculosis* strains across regions can reveal outbreaks of more virulent strain types, including Beijing strains [7].

Genome sequencing of *M. tuberculosis* has gained traction for both clinical and epidemiological investigations. These applications have provided insights into mutations underlying drug resistance, circulating strain types and virulence and transmission dynamics, thereby with the potential to inform clinical and surveillance activities. New genomic tools allow for whole-genome sequencing (WGS) with increasing opportunities to use it directly from the sputum [8]. Together with new analysis methods, WGS data can be used to profile the bacteria for drug resistance [5, 9, 10], characterize ancient and modern lineages and different strain types [11], and establish who may have transmitted to whom; thus allow targeted resources to hotspot areas to reduce transmission [12]. These genomic insights are facilitated through advances in health informatics [13].

WGS opportunities are set to revolutionize the diagnosis and clinical management of TB patients, with routine pathogen genetic characterization applied in the UK healthcare system. Building on this success and the recent COVID-19 experience, an increasing number of countries worldwide is seeking to adopt genomics as part of clinical care [14]. However, to be effective for global disease control and maximize impact, next-generation sequencing (NGS) platforms need to be applied in high TB burden settings, which may be resource-poor. To achieve the economies of scale and cost reductions in these settings, it is possible to target a high number of genes (e.g. drug-resistance loci) across many samples using an amplicon-based approach on NGS platforms, or focus on the multiplexing of whole genomes if the transmission is important.

Compared to other pathogen genomes, the *M. tuberculosis* genome (size: 4.4Mb) is relatively clonal with no horizontal gene transfer, but was historically challenging to sequence due to its high guanine–cytosine (GC) content and repetitive nature [15]. The Illumina sequencing platform with its paired short reads and low error rates has been employed successfully to analyse almost the entire genome, including drug-resistance loci [10], with highly variable and GC-rich *pe*/*ppe* genes often excluded due to the difficulties in accurately mapping these repetitive regions [16–18]. Recently, sequencing platforms with long reads (>1 kb) have been applied for the construction of reference genomes and analysis of methylated base modifications [19], but are too costly for implementation as a high throughput tool. Our previous work compared the application of the Illumina MiSeq, Ion Torrent Personal Genome Machine (PGM)™ [15] and Oxford Nanopore Technologies (ONT) platforms [13]. We observed higher sequencing error rates on the ONT platforms, but sufficient coverage to call drug-resistant variants [13]. The ONT sequencing platform is portable enabling the characterization of *M. tuberculosis* in remote and field settings and has the potential to perform multiplexing of samples, leading to cost reductions. Future cost-effectiveness is likely by informed decision-making in clinics through personalization of treatments in drug-resistance settings, as well as by determining geographical regions for the optimal targeting of TB surveillance and control activities. To assess the viability of the ONT platform for these applications, we apply the technology to DNA extracted from *M. tuberculosis* isolates. In a paired analysis, we compare the resulting WGS sequence data to those generated on an Illumina platform, finding high concordance in variant calls between methods and the potential to include genomic regions traditionally excluded in our analysis, such as *pe*/*ppe* genes.

## Results

### Coverage

**Figure 1:**
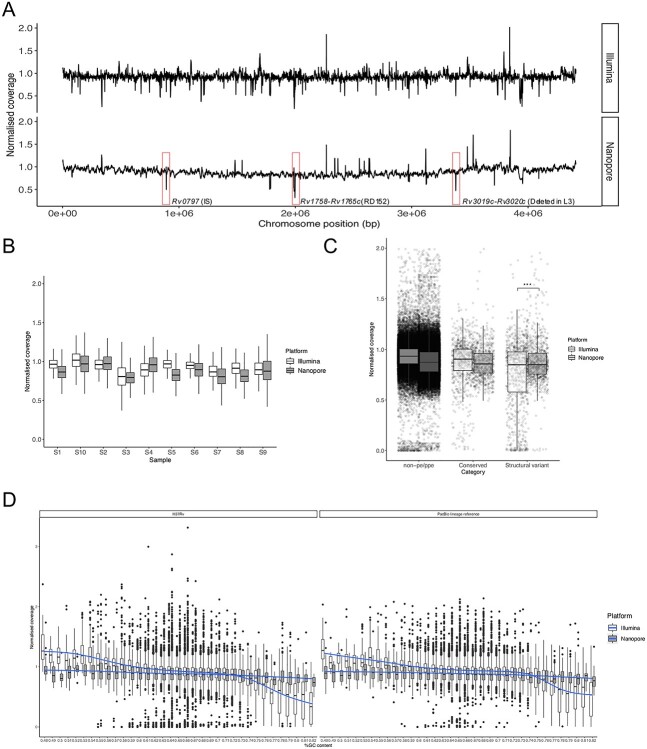
Coverage analysis for Illumina and ONT data. Coverage analysis for ONT and Illumina data across the 10 sample pairs (S1–S10). (**A**) Average median normalized coverage along the chromosome across all samples for both technologies (top Illumina, bottom ONT). Genes with average median coverage <0.5 for ONT platform are annotated: *Rv0797* corresponds to an insertion sequence; *Rv1758-Rv1765c* corresponds to RD152, deleted in L2 and one isolate from L3; and *Rv3019c-Rv3020c* is a genomic region deleted in L3 isolates. The vertical axis shows the median coverage normalized by four housekeeping genes. The horizontal axis shows the position along the chromosome aligned to H37Rv. (**B**) Boxplots of normalized coverage per gene per sample for the 10 pairs. (**C**) Normalized coverage per gene per sample by group as follows: non-*pe*/*ppe* genes, conserved *pe*/*ppe* genes and *pe*/*ppe* genes with structural variants; ^*^^*^^*^ adjusted *P*-value <0.001. (**D**) Normalized coverage distribution per sample per gene by GC content for each sequencing platform. On the left, coverage obtained aligning to H37Rv reference; on the right, coverage obtained aligning to PacBio lineage-specific assemblies.

ONT long reads and Illumina short reads were generated from the sequencing of replicate DNA of 10 clinical isolates originally sourced from Malawi (labelled S1–10; Table S1**,** see Supplementary Data available online at http://bib.oxfordjournals.org/). These isolates covered lineages 1 (L1: 1.1.2, *n* = 1; 1.1.3.2, *n* = 1), 2 (L2: Beijing 2.2.1, *n* = 3), 3 (L3: *n* = 4) and 4 (L4: 4.9, *n* = 1). Sequencing with the ONT platform yielded a median of 67 939 reads per sample, with a median read length of 3806 bp. Illumina data (median number of reads: 1 687 571; read length: 75–100 bp) were generated for the same samples. Mapping to the reference genome (H37Rv GCA_000195955.2) led to a high depth of coverage for all samples (average depth of coverage: Illumina 93.6-fold, ONT 72.2-fold) (Table 1). For all samples, median coverage normalized by four housekeeping genes (*gyrB*, *gyrA*, *rpoB* and *rpoC*) was investigated genome-wide (Figure 1A). Overall, across sample pairs and sequencing platforms, there was high normalized read depth with medians above 0.75 (Figure 1B). Normalized coverage levels in ONT data below 0.5 coincided with lineage-specific deleted regions, including known regions of difference (e.g. RD152 in lineage 2). The presence of these deletions in specific lineages was independently validated in high-quality PacBio whole-genome assemblies [19].

**Table 1:** Summary of 10 sample pairs (S1–S10) sequenced using Illumina and Oxford Nanopore Technology platforms

**Sample**	**Lineage**	**Platform**	**Mean Read Length**	**Number of Reads**	**% Reads Mapped**	**Mean Depth**	**No. of SNPs** ^**a**^
S1	3	ONT	4496	97 949	95.77	94	1144
		Illumina	100	2 000 955	99.39	78	1146
S2	3	ONT	5421	75 742	95.79	87	1154
		Illumina	100	1 593 992	99.52	67	1157
S3	3	ONT	4204	113 137	97.45	102	1158
		Illumina	75	11 239 186	99.32	251	1160
S4	3	ONT	4784	72 196	96.49	74	1156
		Illumina	75	6 929 436	99.31	152	1158
S5	4.9	ONT	6958	46 188	94.49	69	259
		Illumina	100	1 320 558	99.78	55	259
S6	1.1.2	ONT	4997	60 416	95.63	64	1741
		Illumina	100	2 116 280	99.35	90	1746
S7	1.1.3.2	ONT	4411	75 431	96.81	72	1763
		Illumina	100	1 127 055	99.22	48	1772
S8	2.2.1	ONT	4296	63 528	96.98	59	1154
		Illumina	100	1 334 916	99.53	55	1158
S9	2.2.1	ONT	5395	43 239	97.29	51	1154
		Illumina	100	1 781 150	99.46	76	1158
S10	2.2.1	ONT	3468	63 682	97.78	48	1115
		Illumina	100	1 510 044	99.57	65	1119

^a^High-quality SNPs obtained at an alternate frequency of 0.7.

Through the mapping of the ONT data against a representative PacBio assembly for each lineage, high normalized coverage was achieved genome-wide. There were several peaks with normalized coverage below 0.5 belonging to insertion sequences (e.g. IS*6110*) or deleted genes in specific strains (e.g. RD152 region in sample S1) (Figure S1, see Supplementary Data available online at http://bib.oxfordjournals.org/). Overall, these results suggest that ONT technology has performed well, including in repetitive regions. The genes with the lowest coverage in Illumina data were mostly *pe*/*ppe* genes, whose mapping accuracy with short reads is known to be low [17, 18], due to their high GC content and repetitive regions. For the 85 *pe*/*ppe* genes thought to be non-conserved harbouring structural variants that disrupt their protein sequences [16], there was lower sequencing coverage in Illumina compared to ONT data (*t*-test adjusted *P*<0.001, Figure 1C).

Although aligning to a lineage-specific reference improved the coverage for Illumina (Figure S1, see Supplementary Data available online at http://bib.oxfordjournals.org/), extreme GC content disproportionately reduced coverage in short read compared to long-read data (Figure 1D). Genes with the lowest average values of normalized coverage in Illumina data had higher coverage in ONT data (*t*-test adjusted *P* < 0.001, Figure S2, see Supplementary Data available online at http://bib.oxfordjournals.org/). Two genes had greater coverage in Illumina compared to ONT data in L2 and L3 sample pairs, coinciding with an insertion sequence (*Rv0797*) and a conserved hypothetical protein (*Rv1765c*). The latter belongs to RD152, which was deleted in all L2 isolates and one L3. However, due to the high similarity (97%) between *Rv1765c* and *Rv2015c* sequences, the Illumina platform seems to not capture the deletion.

### Variant calling

Variants were called using Freebayes software retaining all sites where at least one sample had >50% alternate reads, leading to 9052 unique positions. For the analysis, Illumina variants with an allele depth (AD) fraction of at least 0.7 were considered true variants. Due to the high error rate of ONT sequencing, almost all positions at which a true variant exists contain a mixture of alternate and reference alleles. To find the optimum cut-off, which balances the sensitivity (true positive rate) and specificity (true negative rate), alternate-allele proportions for each site in the ONT replicates were compared to their Illumina counterparts. An optimum alternate-allele proportion value of 0.7 was chosen, keeping the true positive rate > 97% and true negative rate > 91%, and the false-positive rate < 1% (Figure S3, see Supplementary Data available online at http://bib.oxfordjournals.org/). After refining genotype calls using the chosen minimum alternate frequency of 0.7 and removing repetitive and poorly covered regions in Illumina alignments, a final filtered dataset of 3955 SNPs covering >89% of the genome was retained for subsequent analysis (Figure S4, see Supplementary Data available online at http://bib.oxfordjournals.org/). The chosen frequency cut-off of 0.7 was validated using ONT sequence data for four replicates of the H37Rv reference strain [13]. After implementing the pipeline discussed earlier, there was a high concordance between the H37Rv replicates, with only 4 discrepancies found among the 29 SNPs identified.

The concordance of SNPs and small indels detected by ONT and Illumina data was assessed. For all pairs, >99% of the total SNPs identified were called in both samples, showing few combined platform discrepancies (median 3.5; range: 0–9 SNPs) (Table 2). Agreement between platforms for depth of coverage and alternate frequencies was assessed. Good coverage in Illumina coincided with good coverage in ONT, and the alternate frequencies were observed to be lower in ONT than in Illumina, consistent with the noisier nature of the ONT technology (Figure S5, see Supplementary Data available online at http://bib.oxfordjournals.org/). Most discrepancies arose in the few SNPs called in Illumina but not in ONT data, due to alternate frequency values just below the 0.7 AD cut-off (Table S2, see Supplementary Data available online at http://bib.oxfordjournals.org/). In addition, every sample except S5 (L4.9) differed in the call for the (H37Rv) genomic position 55 553. This position is in a GC-rich region where ONT data had a CCG (3 nucleotide) insertion followed by a nucleotide change (C - > T), whereas the variant called in Illumina data only included the SNP. The multiple CCG repeats present in the sequence, led to the Illumina data analysis not capturing the insertion. Additionally, ONT data for sample S10 showed an SNP in a GC-rich region whilst in its Illumina counterpart, it was identified as a 1 bp insertion followed by the SNP, suggesting an error in the ONT call.

**Table 2:** Concordance of variants found using Illumina and Oxford Nanopore Technology (ONT) platforms

**Sample Pair**	**SNPs**			**Small Indels**			**Large Structural Variants** ^**a**^		
	**ONT only**	**Illumina only**	**Both**	**ONT only**	**Illumina only**	**Both**	**ONT only**	**Illumina only**	**Both**
S1	0	2	1144	3	4	94	58	9	20
S2	0	3	1154	3	8	88	64	6	17
S3	0	2	1158	5	7	84	62	6	14
S4	0	2	1156	4	7	84	66	4	14
S5	0	0	259	2	2	28	14	0	4
S6	0	5	1741	0	9	115	67	5	20
S7	0	9	1763	3	5	108	61	6	19
S8	0	4	1154	3	6	97	68	8	14
S9	0	4	1154	2	7	95	67	9	16
S10	1	5	1114	2	5	97	72	12	18

^a^Includes indels > 15 bp.

The majority (>87%) of small indels called at an alternate frequency of 0.7 were correctly captured by both platforms (Table 2). However, more discrepancies were identified with small indels than with SNPs (median 9; range 4–12 small indels). These discrepancies were mostly driven by small indels (1 bp) in polyC/polyG repeats, which were called from Illumina but not in ONT sequence data (Table S3, see Supplementary Data available online at http://bib.oxfordjournals.org/). On the other hand, the second type of calls in ONT that differed from Illumina was larger indels (8–10 bp), in which the AD fraction in Illumina was slightly lower than 0.7, suggesting that these larger variants called by ONT were not spurious (Table S4, see Supplementary Data available online at http://bib.oxfordjournals.org/). Larger structural variants (>15 bp) were investigated with Delly software. Long reads allow more accurate identification of large indels. As expected, a higher number of large variants was observed in ONT (median 81) compared to Illumina (median 24) data (Table 2), with deletions having the highest agreement between platforms (pairwise sample overlap: median 17, range 2–20 large indels).

### Strain typing and phylogenetics

**Figure 2:**
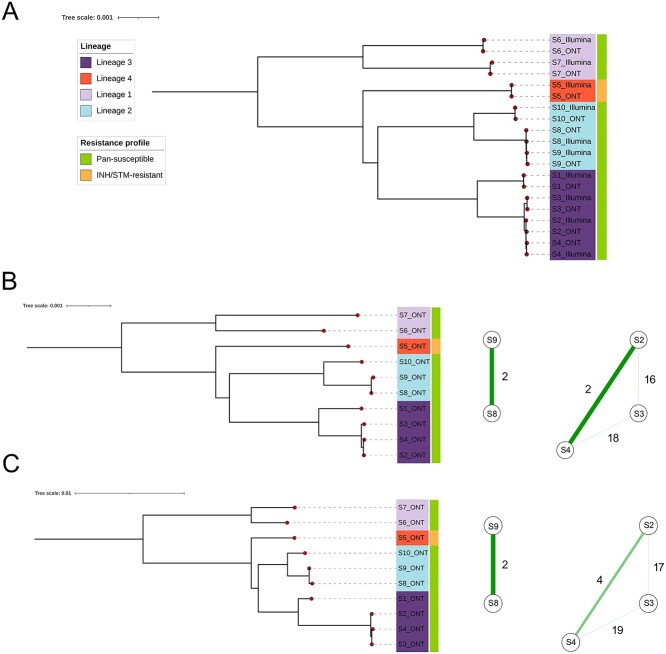
Phylogenetic trees and transmission networks. Maximum likelihood phylogenetic trees and transmission networks for the samples studied. Isolates are coloured by lineage. Drug-resistance profile obtained by phenotypic drug susceptibility testing is shown in the strip labels on the trees. (**A**) Phylogenetic tree reveals high degree of concordance and clustering of replicates sequenced using ONT and Illumina platforms, reconstructed with 3955 SNPs excluding genomic repetitive regions. (**B**) Phylogenetic tree of ONT sequenced samples using the 3955 SNPs, as well as transmission networks for lineage L2 (S8 and S9) and L3 (S2, S3 and S4) clusters showing SNP distances. (**C**) Phylogenetic tree of ONT sequenced samples using the 3955 SNPs in addition to 568 more polymorphic sites located in *pe*/*ppe* genes, as well as transmission networks for lineage L2 (S8 and S9) and L3 (S2, S3 and S4) clusters with SNP distances.

Lineage prediction was performed by TB-Profiler software using the 3955 high-quality SNPs covering >89% of the genome, and consistency between pairs was assessed. All predictions were found to be identical between Illumina and ONT platforms confirming the robust nature of the variant calling process (Table 1). To further investigate the use of the ONT platform to perform clustering, phylogenetic reconstruction was performed using IQ-TREE software. Clear clustering of strain types was observed with long internal branches separating each major lineage. In addition, each sample pair formed a monophyletic clade with short terminal branch lengths indicating the near-identical pattern of variation detected through both platforms (Figure 2; Figure S6, see Supplementary Data available online at http://bib.oxfordjournals.org/). Two and three samples belonging to L2 (S8, S9) and L3 (S2, S3, S4), respectively were closely related, with the number of SNP differences below or equal to 20.

To increase the accuracy of the phylogenetic reconstruction, potentially for transmission analysis, base calls were manually curated, and SNPs which were called as reference with alternate AD frequencies between 0.6 and 0.7 were redesignated as alternate base calls. Following this, the reconstruction of the phylogenetic tree with only ONT isolates was performed using the 3955 polymorphic sites (Figure 2**;**Figure S6, see Supplementary Data available online at http://bib.oxfordjournals.org/). Samples within a putative L2 transmission cluster (S8 and S9) differed by 2 SNPs, whilst the distance within the L3 transmission cluster (S2, S3, S4) varied between 2 and 18 SNPs. Characterization of transmission chains is of epidemiological importance, and due to the small numbers of variants that sometimes separate closely related isolates, accurate estimation of the number of SNPs differences between samples is crucial. Previous studies have shown how long-read sequencing solves some of the traditional Illumina blind spots [20], including the successful assembly and variant calling of *pe*/*ppe* genes with ONT data [21]. On this basis, 150 out of 169 *pe*/*ppe* genes with good coverage (>0.7 normalized mean coverage) were included to complement the genomic regions analysed and therefore potentially achieve a deeper separation of the transmission clusters. These regions overlapped with previous studies [16, 22]. An extra 568 high-quality SNPs were added, resulting in one additional SNP within the transmission cluster from L2 (S8, S9) and four extra SNPs for L3 (S2, S3, S4), thereby slightly increasing the differences obtained within highly similar samples (Figure 2C).

### Drug-resistance prediction

Drug-resistance profiles were predicted by TB-Profiler software using the filtered set of 3955 SNPs. Predictions were compared across replicates and matched perfectly between platforms, leading to nine pan-susceptible isolates and one pre-MDR isolate. In addition, identical variants were found across the 42 genes analysed by the TB-Profiler. Drug susceptibility test data was used to confirm these predictions with all matching, except one (Table S1, see Supplementary Data available online at http://bib.oxfordjournals.org/). One inconsistency was observed in the pre-MDR isolate (sample S5), where although isoniazid resistance was genotypically and phenotypically concordant (*katG* S315T present in both ONT and Illumina data), streptomycin resistance was observed through drug susceptibility testing but not in the genotypic prediction. Upon further inspection of non-associated variants in streptomycin-resistance genes in isolate S5, a premature stop codon was observed (in both Illumina and ONT data) in *gid* (S136^*^), which is the likely explanation for the discrepancy between phenotypic and genotypic predictions.

## Discussion

The benefits of using WGS technologies in clinical and epidemiological settings, such as the characterization of transmission networks, or for detection of drug resistance-associated mutations to inform treatment decisions, have been described [12, 13]. Nevertheless, the associated costs of WGS can limit their application, especially in remote, field or resource-poor settings. The recent development of portable sequencing devices powered by laptops, such as ONT MinION, is significantly reducing the costs and infrastructure necessary for sequencing, thereby improving accessibility [23, 24]. This accessibility would be useful for infection control in the high TB transmission setting of the Karonga District, Malawi, the source of our samples. In parallel, the possible direct sequencing from sputum samples has been successfully reported, taking up to 5 days [8, 23, 25], which will shorten the time from specimen collection to a drug-resistance profile, leading to timely and personalized treatment that can be significantly delayed when culture isolation is required (up to 3 weeks).

To assess the performance of Illumina short-read and ONT long-read platforms, we have carried out a comparative analysis of 10 sample pairs with data from both technologies. Illumina technology with a low sequencing error rate is considered the gold standard and therefore has been applied to inform on drug resistance or transmission, but the performance of ONT, with its known higher error rate, is less clear. Several studies have evaluated the performance of ONT sequencing in target-sequencing approaches for drug-resistance detection [25–27], finding good concordance between Illumina and ONT, or in WGS analysis [28]. For ONT sequencing data, an even coverage distribution along the chromosome was observed, with drops coinciding with deleted genes or regions, such as RD152 (*Rv1758c-Rv1765c*) in L2, or insertion sequences, whose presence/absence is variable among different strains. Coverage levels were not dependent on GC content, with high values even in the extremely GC-rich genes (>80% GC content). Using a lineage-specific genome as a reference yielded an expected overall improvement in coverage across both platforms. However, Illumina replicates of L3 isolates still failed to reach similar values to those of ONT in the high GC content regions, revealing the higher susceptibility of the short-read sequencing platform to GC-rich genes. Blind spots for Illumina sequencing technologies have been previously reported [18], for which long-read sequencing technologies can assist [20, 21]. In accordance with previous work [21], our study demonstrates that long-read data has the potential to elucidate complex regions, such as *pe*/*ppe* genes, which due to their GC-rich and repetitive nature have been systematically excluded from WGS analysis, losing potential phylogenetic information [16, 22]. Coverage of the Illumina replicates on these regions, and more specifically in the most diverse genes of these two families, was shown to be significantly lower than their ONT counterparts, supporting the potential inclusion of these genes for the downstream analysis in WGS from ONT. This could assist with understanding the genetic diversity of *pe/ppe* genes, whose functions are largely still unknown, but some are involved in host–pathogen interactions and thereby promising targets for vaccine development [16].

The performance of the variant calling pipeline for ONT sequences was investigated and compared to the Illumina data, considering the latter as a gold standard. The ONT platform is prone to sequencing errors, whereas Illumina’s high sequencing accuracy makes it preferred for the identification of SNPs and small indels [15]. In contrast, larger structural variants are difficult to capture with short reads, thus applying a hybrid approach involving the assembly of long reads with correction using short reads can improve the accuracy and completeness of variant detection. For the evaluation of the variant calling method in ONT data, an alternate AD fraction ≥0.7 was determined as the optimal cut-off based on the true and false-positive error rates. The exclusion of repetitive regions (e.g. *pe*/*ppe* genes) led to good agreement between platforms for SNPs and small indels, as previously shown in other work [25], with discrepancies often being found at an AD between 0.6 and 0.7, suggesting the potential use of the lower cut-off of 0.6 to include more true positive calls. With SNPs covering more than 89% of the genome, an accurate phylogenetic reconstruction was obtained, supporting the utility of ONT for variant identification and lineage profiling. Moreover, the inclusion of 150 *pe*/*ppe* genes with high levels of coverage, which would normally be among the regions excluded, added extra variants that have the potential of being phylogenetically informative. The possibility of including extra variants may lead to an improved resolution that would be of special interest in outbreak settings, where transmission analysis of closely related isolates can be potentially better established.

One of the most important applications of the ONT MinION portable device is the accurate detection of drug-resistant variants, which can inform and assist patient management in a timelier manner than traditional phenotypic tests. A promising cost-effective approach to the high throughput evaluation of drug-resistant loci in clinical isolates is target-amplicon sequencing [29]. We validated the high quality of the variant calling process on ONT data for 42 known *M. tuberculosis* drug-resistant loci, finding congruent results with their Illumina counterparts. This outcome suggests the potential identification of drug-resistant variants from ONT data, including within a target-amplicon framework.

Limitations of the study include the low number of isolates analysed, the low intra-lineage diversity and a limited number of drug-resistant isolates. Whilst the latter may limit the investigation of variants in drug resistance-associated loci, given the error rate of ONT including within these loci, our approach robustly characterizes the sequences of drug-resistance genes. It is thus reasonable to expect that the approach will also accurately characterize the sequence of genes that contain variants and, by extension, predict resistance. Previous work has shown accurate drug resistance variant detection through different methods [25, 28], with promising results for its use for diagnostics purposes in the clinic. However, for the complete reliance on *in silico* drug resistance prediction based on genotypes, an improved understanding of catalogue of resistance mutations is essential. A more complete characterization of phenotype–genotype associations for certain drugs is required and the phenotypic–genotypic inconsistency observed in this analysis reflects this need. WGS facilitates a more comprehensive analysis compared to targeted gene sequencing. The use of long reads can cover repetitive regions of the genome, and thereby help elucidate compensatory or epistatic mutations that could be crucial for a better understanding of drug-resistance mechanisms in *M. tuberculosis*.

In conclusion, the data obtained through this analysis supports the use of ONT sequencing platforms for the detection of well-established drug resistance variants and phylogenetic reconstruction, with potential application in transmission analysis, since the underpinning SNP variant calling process appears robust. However, due to the high error rate, Illumina remains the best option for small indel analysis, suggesting that for their accurate study with ONT data, a hybrid correcting approach is warranted. Moreover, we demonstrate the possibility of including additional genomic regions in the standard variant calling pipelines, such as the *pe*/*ppe* genes, which due to their implications in pathogenicity and host–pathogen interactions could give insights into epidemiological implications, as well as potentially improve the resolution of transmission clusters. Furthermore, for variants in more complex gene arrangements that might fail to be captured using the H37Rv reference, the use of lineage-specific reference genomes could be practical. The portable MinION technology could therefore be implemented and is likely to gain traction for epidemiological, phylogenetic or drug resistance detection applications, providing much-needed assistance in the control of TB, especially in high-burden settings where impacts will be greater.

## Methods

### Culture, DNA extraction and sequencing

The 10 isolates analysed in this study were sourced from the TB patients in Karonga (Malawi) between 2001 and 2009, with isolates stored at the London School of Hygiene and Tropical Medicine. The bacterial culture and extraction of genomic DNA were carried out at the LSHTM Biosafety Level 3 containment facility. Briefly, *M. tuberculosis* isolates were pre-cultured in Middlebrook 7H9 supplemented with 0.05% Tween 80 and 10% albumin-dextrose-catalase at 37°C to mid-log phase. Once reached exponential growth, they were passaged to roller bottles until desired optical density (OD = 0.6–0.8). Heat inactivation (1 h at 80°C) followed by the Cetyl trimethyl ammonium bromide (CTAB)-chloroform-isoamyl alcohol method was used for genomic DNA extraction [30]. WGS of DNA samples was performed with ONT (MinION Flow Cell with R10.3 nanopore chemistry; SQK-LSK109 ligation-based sequencing kit) and Illumina HiSeq 4000 (150 bp paired-ends) platforms through The Applied Genomics Centre at LSHTM. A further set of four DNA replicates for the reference H3Rv strain also underwent sequencing using the ONT MinION platform. All raw sequencing data are available; see Table S1 (see Supplementary Data available online at http://bib.oxfordjournals.org/) for accession numbers.

### Bioinformatics pipeline

Base-calling of ONT raw sequence data was performed with the bonito base caller (model dna_r9.4.1_e8.1_sup@v3.3) [31] and reads aligned to the H37Rv reference genome (GCA_000195955.2) using minimap2 (v2.17-r941) software [32] discarding ambiguous reads. Depth of coverage along the chromosome and median coverage per annotated gene was calculated with BEDTools (v2.29.2) [33], using the alignments of data obtained by ONT and Illumina platforms. To compare between samples, median coverage per gene per sample was normalized by the coverage of four housekeeping genes (*gyrB*, *gyrA*, *rpoB* and *rpoC*). These genes are known to not be deleted or duplicated and expected to have a good ‘average’ coverage. Lineage-specific reference genomes were selected among high-quality PacBio assemblies [19] and used to assess levels of coverage. Due to the high error rate of the ONT platform, a mixture of alternate and reference alleles is often found. To identify an optimum cut-off for variant calling, a minimum alternate allele frequency of 0.5 was used in the variant calling process carried out using Freebayes (v1.3.2) software [34]. Variant calls obtained in Illumina data with an allele frequency of 0.7 were considered as true variants. Alternative allele frequency cut-off values of 0.5, 0.6, 0.7, 0.8, 0.9 and 1.0 for ONT variant calls were used and true and false positive and negative rates for each of the cut-offs were calculated. True- and false-positive rates were compared and evaluated using a receiver operator characteristic curve analysis. A final cut-off of 0.7 was determined to perform variant calling and validated using ONT data from the H37Rv replicates.

To obtain a curated set of SNPs for the subsequent analysis, variants were filtered (Figure S4, see Supplementary Data available online at http://bib.oxfordjournals.org/). In brief, regions with repetitive sequences that generate mapping problems (GitHub repository https://github.com/pgomezgonzalez/nanopore_tb_data_analysis), such as *pe*/*ppe* genes or insertion sequences, were excluded, and only SNPs were selected. Genotype calls were defined by read depth (DP) and alternate AD fraction, with a minimum DP of 10 required to determine a position and an AD ≥ 0.7 needed to retain the alternate call. The resulting refined SNP dataset was used for the agreement evaluation between sample pairs and their phylogenetic reconstruction. Small indels called using Freebayes (v1.3.2) were filtered using the same pipeline as SNPs. Delly (v0.8.7) software [35] was used for large structural variants (indels with size >15 bp). Lineage- and drug-resistance profiling of the sample pairs was carried out with TB-Profiler software (v3.0, commit version: de4e796) [13]. Maximum likelihood phylogenetic reconstruction of the genomes was performed with IQ-TREE (v1.6.12) with a GTR + G + ASC nucleotide substitution model [36] by using genome-wide SNPs excluding repetitive regions or including the 150 *pe*/*ppe* genes with good coverage and visualized together with annotations in iTOL software. Custom scripts used in the analysis pipeline are available in a GitHub repository (https://github.com/pgomezgonzalez/nanopore_tb_data_analysis).

**Key Points**

- Robust variant calling following sequencing of Oxford Nanopore Technologies (ONT).
- Suitability of ONT sequencing to detect variants in drug resistance-associated loci.
- Enhanced transmission analysis by deeper resolution from long-read sequence data.

## Data availability

Raw sequencing data are available from the European Nucleotide Archive; see Table S1 (see Supplementary Data available online at http://bib.oxfordjournals.org/) for a list of accession numbers.

## Authors’ contributions

S.C., J.E.P. and T.G.C. conceived and directed the project. P.J.G.-G. undertook sample processing and DNA extraction. P.J.G.-G. also performed bioinformatics and statistical analyses under the supervision of S.C., J.E.P. and T.G.C. P.J.G.-G., S.C., J.E.P. and T.G.C. interpreted results. P.J.G.-G. wrote the first draft of the manuscript with inputs from J.E.P. and T.G.C. All authors commented and edited on various versions of the draft manuscript and approved the final manuscript. P.J.G.-G., J.E.P. and T.G.C. compiled the final manuscript.

## Funding

MRC-LID Ph.D. studentship (to P.J.G.-G.); Newton Institutional Links Grant (British Council, No. 261868591 to J.E.P.); Medical Research Council UK (grant nos. MR/M01360X/1, MR/N010469/1, MR/R025576/1 and MR/R020973/1 to T.G.C.); BBSRC (grant no. BB/R013063/1); Medical Research Council UK (grant nos. MR/M01360X/1, MR/R025576/1 and MR/R020973/1 to S.C.).

## Ethics approval and consent to participate

The studies were approved by the Health Sciences Research Committee in Malawi (#424) and by the LSHTM Ethics Committee (#5067). Informed written consent was sought and obtained for all patients in the original study.

## Supplementary Material

Supplementary_revised_bbac256

## Author Biographies

**Paula Gómez-González** has a background in pharmacy, and recently completed a PhD in tuberculosis genomics.
