# Supplementary material for: Portable sequencing of Mycobacterium tuberculosis for clinical and epidemiological applications
Source: Brief Bioinform. 2022 Jul 27;23(5):bbac256. doi: 10.1093/bib/bbac256 (PMC9487601; doi:10.1093/bib/bbac256)
Supplement: Supplementary_revised_bbac256 [file supplementary_revised_bbac256.docx]

**Portable sequencing of *Mycobacterium tuberculosis* for clinical and epidemiological applications**

Paula J. Gómez-González^1^

Susana Campino^1^

Jody Phelan^1,*^

Taane Clark^1,2,*^

^1^ Faculty of Infectious and Tropical Diseases, London School of Hygiene & Tropical Medicine, WC1E 7HT London, UK

^2^ Faculty of Epidemiology and Population Health, London School of Hygiene & Tropical Medicine, WC1E 7HT London, UK

* Joint authors

** correspondence

taane.clark@lshtm.ac.uk

Department of Infection Biology,

Faculty of Infectious and Tropical Diseases

London School of Hygiene & Tropical Medicine, Keppel Street, London, UK

**Briefings in Bioinformatics, Case Study**

**Additional File**

**Table S1. ENA accession number for study samples undergoing sequencing using Illumina and Oxford Nanopore Technology (ONT) platforms.**

| Sample | Lineage | Drug resistance genotypic profile | Phenotypic DST profile | Illumina Sequencing | ONT |
| --- | --- | --- | --- | --- | --- |
| S1 | 3 | Pan-susceptible | Pan-susceptible | ERR161062 | ERR8170869 |
| S2 | 3 | Pan-susceptible | Pan-susceptible | ERR182032 | ERR8170870 |
| S3 | 3 | Pan-susceptible | Pan-susceptible | ERR245682 | ERR8170871 |
| S4 | 3 | Pan-susceptible | Pan-susceptible | ERR245678 | ERR8170872 |
| S5 | 4.9 | INH resistant | INH and STR resistant | ERR181826 | ERR8170873 |
| S6 | 1.1.2 | Pan-susceptible | Pan-susceptible | ERR181951 | ERR8170874 |
| S7 | 1.1.3.2 | Pan-susceptible | Pan-susceptible | ERR181929 | ERR8170875 |
| S8 | 2.2.1 | Pan-susceptible | Pan-susceptible | ERR181821 | ERR8170876 |
| S9 | 2.2.1 | Pan-susceptible | Pan-susceptible | ERR221538 | ERR8170877 |
| S10 | 2.2.1 | Pan-susceptible | Pan-susceptible | ERR221573 | ERR8170878 |
| H37Rv | 4 | Pan-susceptible | Pan-susceptible | - | ERR8441303, ERR8441304, ERR8441305, ERR8441306 |

INH = Isoniazid, STR = Streptomycin; DST = drug susceptibility testing

**Table S2. Discrepancies between Illumina and Oxford Nanopore Technology (ONT) SNP calls.**

| Sample pair | POS REF | Gene | ONT alternative allele (depth*) | Illumina alternative allele (depth*) |
| --- | --- | --- | --- | --- |
| S1 | 55553 CCG | *Rv0050* | TCG (0.37)  CCGTCG (0.60) | **TCG (0.88)** |
|  | 1608276 A | *Rv1431* | C (0.69) | **C (1)** |
| S2 | 55553 CCG | *Rv0050* | TCG (0.44)  CCGTCG (0.53) | **TCG (0.82)** |
|  | 4027914 C | *Rv3586* | T (0.69) | **T (1)** |
|  | 4323831 C | *Rv3849* | T (0.68) | **T (1)** |
| S3 | 55553 CCG | *Rv0050* | TCG (0.36)  CCGTCG (0.61) | **TCG (0.85)** |
|  | 1608276 A | *Rv1431* | C (0.65) | **C (1)** |
| S4 | 55553 CCG | *Rv0050* | TCG (0.25)  CCGTCG (0.69) | **TCG (0.87)** |
|  | 4027914 C | *Rv3586* | T (0.65) | **T (1)** |
| S6 | 50906 C | *Rv0046c* | T (0.68) | **T (0.98)** |
|  | 55553 CCG | *Rv0050* | TCG (0.06)  CCGTCG (0.92) | **TCG (0.74)** |
|  | 1585283 A | *Rv1409* | C (0.61) | **C (0.98)** |
|  | 1798355 G | *Rv1597* | A (0.61) | **A (1)** |
|  | 2663210 G | *Rv2380c* | A (0.69) | **A (0.96)** |
| S7 | 55553 CCG | *Rv0050* | TCG (0.12)  CCGTCG (0.79) | **TCG (0.82)** |
|  | 1585283 A | *Rv1409* | C (0.60) | **C (1)** |
|  | 1798355 G | *Rv1597* | A (0.64) | **A (1)** |
|  | 2092970 C | *Rv1843c* | T (0.68) | **T (0.98)** |
|  | 2093715 T | *Rv1843c* | C (0.61) | **C (0.97)** |
|  | 2827111 C | *Rv2510c* | T (0.68) | **T (0.94)** |
|  | 3220048 C | *Rv2913c* | T (0.57) | **T (1)** |
|  | 3479561 G | *Rv3111* | A (0.69) | **A (0.99)** |
|  | 3653225 C | *Rv3271c* | T (0.66) | **T (0.91)** |
| S8 | 55553 CCG | *Rv0050* | TCG (0.23)  CCGTCG (0.69) | **TCG (0.83)** |
|  | 460413 C | *Rv0384c* | T (0.65) | **T (0.94)** |
|  | 1831219 CAC | *Rv1629* | CCC (0.19)  CC (0.78) | **CCC (1)** |
|  | 3010993 C | *Rv2693c* | T (0.68) | **T (1)** |
| S9 | 55553 CCG | *Rv0050* | TCG (0.09)  CCGTCG (0.91) | **TCG (0.73)** |
|  | 460413 C | *Rv0384c* | T (0.65) | **T (0.94)** |
|  | 1097220 C | *Rv0981* | T (0.69) | **T (1)** |
|  | 1831219 CAC | *Rv1629* | CCC (0.19)  CC (0.78) | **CCC (1)** |
| S10 | 39030 C | *Rv0035* | T (0.36) | **T (0.80)** |
|  | 55553 CCG | *Rv0050* | TCG (0.20)  CCGTCG (0.74) | **TCG (0.73)** |
|  | 549361 CGC | *Rv0457c* | **CGG (0.95)**  CGGG (0) | CGG (0.14)  CGGG (0.84) |
|  | 1608276 A | *Rv1431* | C (0.68) | **C (1)** |
|  | 1831219 CAC | *Rv1629* | CCC (0.3)  CC (0.67) | **CCC (1)** |
|  | 4359165 G | *Rv3879c* | C (0.63) | **C (0.99)** |

ONT = Oxford Nanopore Technology; * Allele depth; in bold, platform where alternate allele was called (alternative depth ≥ 0.7; alleles with indels not considered). Note, there were no discrepancies between calls in Illumina and ONT data for sample S5.

**Table S3. Discrepancies between Illumina and Oxford Nanopore Technology (ONT) indel calls.**

| Sample pair | POS | Gene | ONT alternative allele (depth*) | Illumina alternative allele (depth*) |
| --- | --- | --- | --- | --- |
| S1 | 293628 | *Rv0243* | insC (0.61) | **insC (1)** |
|  | 854252 | *Rv0759c* | delC (0.42)  delCC (0.57) | **delC (1)** |
|  | 1365837 | *Rv1222* | insGG (0.45)  insG (0.40) | **insGG (1)** |
|  | 2320329 | *Rv2062c* | **delC (0.75)** | delC (0) |
|  | 2631009 | *Rv2351c* | insTGCCG (0.47) | **insTGCCG (0.93)** |
|  | 2850856 | *Rv2525c* | **delG (0.71)** | delG (0) |
|  | 3296371 | *Rv2947c* | **insCGCGGCC (0.71)** | insCGCGGCC (0.69) |
| S2 | 293628 | *Rv0243* | insC (0.63) | **delC (1)** |
|  | 691887 | *Rv0592* | insC (0.55) | **insC (0.98)** |
|  | 830868 | *Rv0739* | insCG (0.67) | **insCG (1)** |
|  | 854252 | *Rv0759c* | delC (0.44)  delCC (0.54) | **delC (1)** |
|  | 1365837 | *Rv1222* | insGG (0.37)  insG (0.48) | **insGG (1)** |
|  | 2320329 | *Rv2062c* | **delC (0.80)** | delC (0) |
|  | 2536625 | *Rv2264c* | insGG (0.21) | **insGG (1)** |
|  | 2631009 | *Rv2351c* | insTGCCG (0.42) | **insTGCCG (0.89)** |
|  | 2850856 | *Rv2525c* | **delG (0.82)** | delG (0) |
|  | 3131469 | *Rv2823c* | **insTCGGCGATG (0.85**) | insTCGGCGATG (0.64) |
|  | 3296371 | *Rv2947c* | insCGCGGCC (0.65) | **insCGCGGCC (0.74)** |
| S3 | 125830 | *Rv0107c* | insA (0.68) | **insA (1)** |
|  | 293628 | *Rv0243* | insC (0.65) | **insC (1)** |
|  | 691887 | *Rv0592* | insC (0.50) | **insC (0.98)** |
|  | 854252 | *Rv0759c* | delC (0.58)  delCC (0.38) | **delC (1)** |
|  | 1365837 | *Rv1222* | insG (0.45)  insGG (0.43) | **insG (1)** |
|  | 2536625 | *Rv2264c* | insGG (0.25) | **insGG (1)** |
|  | 2631009 | *Rv2351c* | insTGCCG (0.40) | **insTGCCG (0.73)** |
|  | 2320329 | *Rv2062c* | **delC (0.78)** | delC (0) |
|  | 2850856 | *Rv2525c* | **delG (0.79)** | delG (0) |
|  | 3059811 | *Rv2747* | **delT (1)** | delT (0.04) |
|  | 3059829 | *Rv2747* | **insA (0.92)** | insA (0) |
|  | 3131469 | *Rv2823c* | **insTCGGCGATG (0.90)** | insTCGGCGATG (0.48) |
| S4 | 293628 | *Rv0243* | insC (0.61) | **insC (1)** |
|  | 691887 | *Rv0592* | insC (0.67) | **insC (1)** |
|  | 830868 | *Rv0739* | insCG (0.66) | **insCG (1)** |
|  | 854252 | *Rv0759c* | delC (0.50)  delCC (0.37) | **delC (1)** |
|  | 1365837 | *Rv1222* | insG (0.49)  insGG (0.47) | **insG (1)** |
|  | 2536625 | *Rv2264c* | insGG (0.38) | **insGG (1)** |
|  | 2631009 | *Rv2351c* | insTGCCG (0.39) | **insTGCCG (0.80)** |
|  | 3059811 | *Rv2747* | **delT (0.99)** | delT (0.02) |
|  | 3059829 | *Rv2747* | **insA (0.94)** | insA (0) |
|  | 3131469 | *Rv2823c* | **insTCGGCGATG (0.93)** | insTCGGCGATG (0.52) |
|  | 3296371 | *Rv2947c* | **insCGCGGCC (0.76)** | insCGCGGCC (0.49) |
| S5 | 854252 | *Rv0759c* | delC (0.48)  delCC (0.48) | **delC (1)** |
|  | 2059780 | *Rv1817* | insG (0.26) | **insG (0.97)** |
|  | 2320329 | *Rv2062c* | **delC (0.77)** | delC (0) |
|  | 3190145 | *Rv2880c* | **delC (0.84)** | delC (0) |
| S6 | 125830 | *Rv0107c* | insA (0.69) | **insA (1)** |
|  | 191391 | *Rv0161* | insC (0.2) | **insC (0.95)** |
|  | 293628 | *Rv0243* | insC (0.59) | **insC (0.96)** |
|  | 854252 | *Rv0759c* | delC (0.40)  delCC (0.56) | **delC (0.99)** |
|  | 919284 | *Rv0825c* | insG (0.30) | **insG (0.96)** |
|  | 1365837 | *Rv1222* | insG (0.59)  insGG (0.29) | **insG (1)** |
|  | 2547529 | *Rv2275* | insG (0.58) | **insG (0.97)** |
|  | 2730151 | *Rv2434c* | insC (0.17) | **insC (1)** |
|  | 3723901 | *Rv3337* | insT (0.69) | **insT (0.94)** |
| S7 | 293628 | *Rv0243* | insC (0.64) | **insC (1)** |
|  | 854252 | *Rv0759c* | delC (0.57)  delCC (0.41) | **delC (0.97)** |
|  | 1365837 | *Rv1222* | insG (0.51)  insGG (0.34) | **insG (1)** |
|  | 2090400 | *Rv1841c* | **insCCAACGCCACCG (0.86)** | (0.67, **DP=24) |
|  | 2547529 | *Rv2275* | insG (0.68) | **insG (0.91)** |
|  | 3131469 | *Rv2823c* | **insTCGGCGATG (0.88)** | insTCGGCGATG (0.63) |
|  | 3296371 | *Rv2947c* | **insCGCGGCC (0.70)** | insCGCGGCC (0.68, *DP=22) |
|  | 3723901 | *Rv3337* | insT (0.69) | **insT (0.98)** |
| S8 | 125830 | *Rv0107c* | insA (0.65) | **insA (1)** |
|  | 293628 | *Rv0243* | insC (0.69) | **insC (1)** |
|  | 799136 | *Rv0698* | **delC (0.73)** | delC (0) |
|  | 854252 | *Rv0759c* | delC (0.36)  delCC (0.56) | **delC (0.98)** |
|  | 964001 | *Rv0866* | insG (0.44) | **insG (0.98)** |
|  | 987585 | *Rv0888* | insG (0.22) | **insG (0.98)** |
|  | 1365837 | *Rv1222* | insG (0.38)  insGG (0.43) | **insG (1)** |
|  | 2320329 | *Rv2062c* | **delC (0.71)** | delC (0.02) |
|  | 2850856 | *Rv2525c* | **delG (0.79)** | delG (0) |
| S9 | 125830 | *Rv0107c* | insA (0.68) | **insA (0.98)** |
|  | 293628 | *Rv0243* | insC (0.60) | **insC (1)** |
|  | 854252 | *Rv0759c* | delC (0.4)  delCC (0.6) | **delC (0.98)** |
|  | 964001 | *Rv0866* | insG (0.44) | **insG (0.85)** |
|  | 987585 | *Rv0888* | insG (0.16) | **insG (0.94)** |
|  | 1365837 | *Rv1222* | insG (0.46)  insGG (0.46) | **insG (1)** |
|  | 1753519 | *Rv1549* | insC (0.64) | **insC (1)** |
|  | 2850856 | *Rv2525c* | **delG (0.77)** | delG (0) |
|  | 3131469 | *Rv2823c* | **insTCGGCGATG (0.90)** | insTCGGCGATG (0.69) |
| S10 | 125830 | *Rv0107c* | insA (0.65) | **insA (1)** |
|  | 809840 | *Rv0712* | insC (0.24) | **insC (1)** |
|  | 987585 | *Rv0888* | insG (0.27) | **insG (1)** |
|  | 1365837 | *Rv1222* | insG (0.55)  insGG (0.31) | **insG (1)** |
|  | 2338194 | *Rv2081c* | delC (0.37)  insC (0.06) | **insC (0.93)** |
|  | 2850856 | *Rv2525c* | **delG (0.72)** | delG (0) |
|  | 3131469 | *Rv2823c* | **insTCGGCGATG (0.86)** | insTCGGCGATG (0.67) |

ONT = Oxford Nanopore Technology; *Allele depth; **DP = low total read depth at locus; in **bold**, platform where alternate allele was called (allele depth ≥ 0.7).

**Table S4. Large structural variants* identified in Illumina and Oxford Nanopore Technology (ONT) sequence data**

| **Sample pair** | **Insertions** | | | | **Deletions** | | |
| --- | --- | --- | --- | --- | --- | --- | --- |
|  | **ONT only** | **Illumina only** | **Both** | **ONT only** | | **Illumina only** | **Both** |
| S1 | 41 | 1 | 1 | 17 | | 8 | 19 |
| S2 | 46 | 0 | 0 | 18 | | 6 | 17 |
| S3 | 44 | 0 | 0 | 18 | | 6 | 14 |
| S4 | 46 | 0 | 0 | 20 | | 4 | 14 |
| S5 | 11 | 0 | 0 | 3 | | 0 | 4 |
| S6 | 50 | 2 | 1 | 17 | | 3 | 19 |
| S7 | 47 | 1 | 1 | 14 | | 5 | 18 |
| S8 | 48 | 0 | 0 | 20 | | 8 | 14 |
| S9 | 49 | 0 | 0 | 18 | | 9 | 16 |
| S10 | 52 | 0 | 1 | 20 | | 12 | 17 |

*Insertions and deletions over 15 bp identified using Delly software.

**Figure S1. Genome-wide normalised coverage**

**
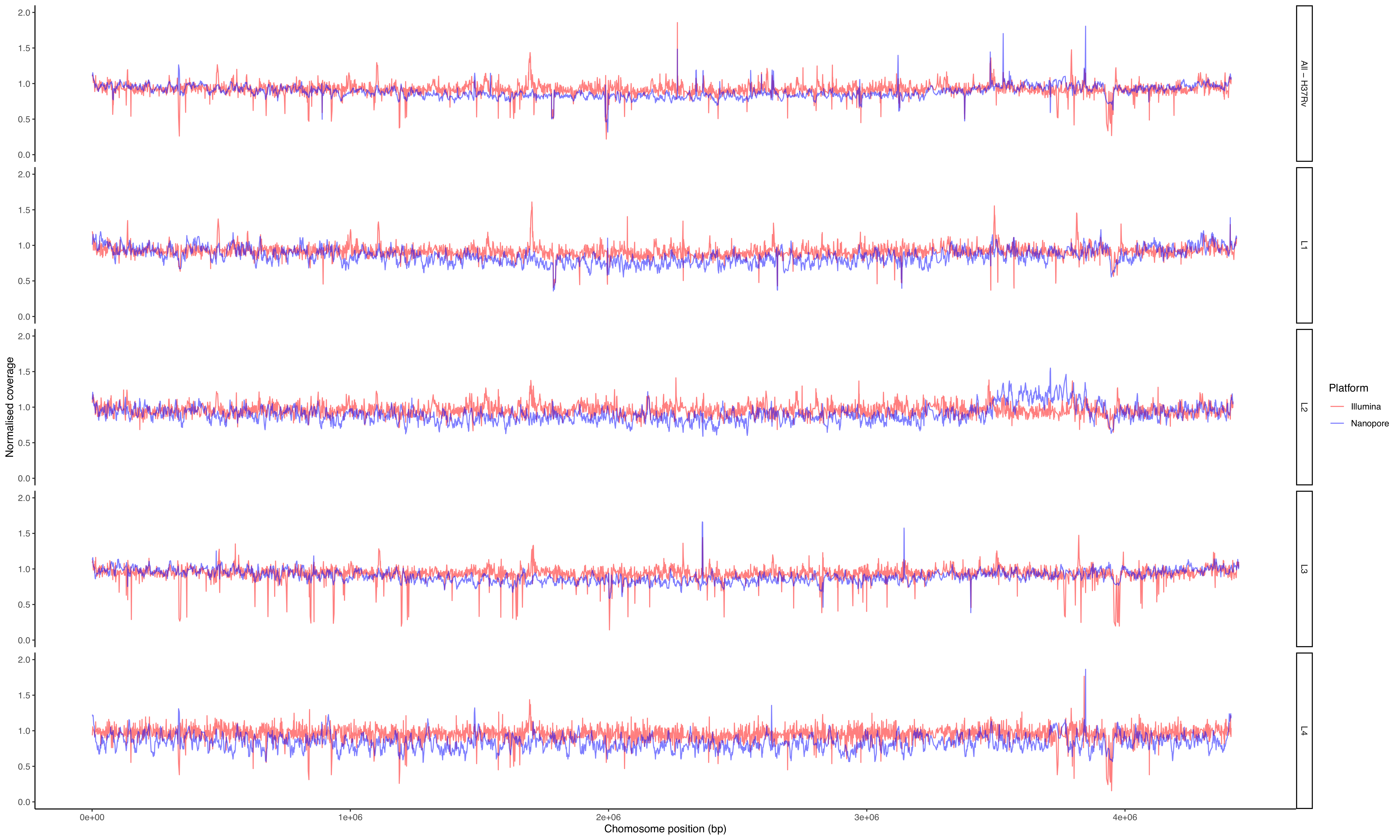
**

Normalised median coverage (vertical axis) along the chromosome (horizontal axis) when using H37Rv as a reference (top track) or PacBio lineage-specific assemblies (second to fifth track (L1-L4). Coverage from Illumina data (red) and ONT data (blue). The large region spanning 3.5 Mbp to 4 Mbp with increased coverage in L2 isolates corresponds to the *DosR* regulon duplication.

**Figure S2. Correlation of normalised coverage between Illumina and Oxford Nanopore Technology (ONT) platforms**

Correlation of normalised median coverage per gene per sample in both sequencing platforms (vertical axis ONT, horizontal axis Illumina) for (**A**) all genes and (**B**) genes with a median normalised coverage < 0.5 in Illumina data in at least one sample. Overall, **(A)** shows a good correlation of coverage between both platforms. In **(B)**, most genes show higher coverage in ONT data. Genes with normalised coverage < 0.1 in both Illumina and ONT represent true deletions. Annotated genes (*Rv0797* and *Rv1765c*) highlight two cases where coverage was higher in Illumina data due to repetitive regions (insertion sequence and highly similarity of a deleted gene belonging to RD152 to *Rv2015c* respectively).

**Figure S3. Receiver-operator characteristic curve for the error rate of Oxford Nanopore Technology (ONT) data**

ROC curve showing the True Positive Rate on the vertical axis with the False Positive Rate on the horizontal axis. All cut-off points studied are annotated in the curve.

**Figure S4. Analysis pipeline**

Summary pipeline of the variant calling, filtering and genotype refining steps carried out to obtain a set of high-quality SNPs. DP = read depth at a locus; AD = alternate allele depth fraction; indels = insertions and deletions.

**Figure S5. Depth of coverage and alternate allele depth fraction correlation between Illumina and Oxford Nanopore Technology (ONT) for SNPs called in both platforms.**

Correlation plots showing (**A**) read depth (RD) and (**B**) alternate allele depth fraction (AF) between Illumina and ONT for positions with SNPs called in both platforms, with Illumina on the horizontal axis and ONT on the vertical axis. **(A)** shows a good correlation between read depth in positions with concordant SNPs; **(B)** shows how ONT reads are noisier than the corresponding Illumina reads, with the fraction of alternate allele depth for ONT with lower values (0.7-1) than the Illumina platform (>0.92).

**Figure S6. Cladogram of Oxford Nanopore Technology (ONT) and Illumina sequenced isolates**

Cladogram representing the branching order with equal branch lengths for the 10 pairs of Illumina and ONT isolates **(A)** and only the 10 ONT isolates **(B)**; INH = Isoniazid, STR = Streptomycin.
